# Generation of hemipelvis surface geometry based on statistical shape modelling and contralateral mirroring

**DOI:** 10.1007/s10237-022-01594-1

**Published:** 2022-06-17

**Authors:** Praveen Krishna, Dale L. Robinson, Andrew Bucknill, Peter Vee Sin Lee

**Affiliations:** 1 Department of Biomedical Engineering, University of Melbourne, Melbourne, VIC 3010 Australia; 2 Department of Orthopaedic Surgery, Royal Melbourne Hospital, Parkville, VIC Australia; 3 Department of Surgery, University of Melbourne, Melbourne, VIC Australia

**Keywords:** Statistical shape modelling, Contralateral mirroring

## Abstract

Personalised fracture plates manufactured using 3D printing offer an improved treatment option for unstable pelvic ring fractures that may not be adequately secured using off-the-shelf components. To design fracture plates that secure the bone fragments in their pre-fracture positions, the fractures must be reduced virtually using medical imaging-based reconstructions, a time-consuming process involving segmentation and repositioning of fragments until surface congruency is achieved. This study compared statistical shape models (SSMs) and contralateral mirroring as automated methods to reconstruct the hemipelvis using varying amounts of bone surface geometry. The training set for the geometries was obtained from pelvis CT scans of 33 females. The root-mean-squared error (RMSE) was quantified across the entire surface of the hemipelvis and within specific regions, and deviations of pelvic landmarks were computed from their positions in the intact hemipelvis. The reconstruction of the entire hemipelvis surfaced based on contralateral mirroring had an RMSE of 1.21 ± 0.29 mm, whereas for SSMs based on the entire hemipelvis surface, the RMSE was 1.11 ± 0.29 mm, a difference that was not significant (*p* = 0.32). Moreover, all hemipelvis reconstructions based on the full or partial bone geometries had RMSEs and landmark deviations from contralateral mirroring that were significantly lower (*p* < 0.05) or statistically equivalent to the SSMs. These results indicate that contralateral mirroring tends to be more accurate than SSMs for reconstructing unilateral pelvic fractures. SSMs may still be a viable method for hemipelvis fracture reconstruction in situations where contralateral geometries are not available, such as bilateral pelvic factures, or for highly asymmetric pelvic anatomies.

## Introduction

The increased availability of metallic 3D printing systems has motivated considerable interest in the manufacture of personalised fracture plates for treatment of bone fractures in clinical settings (Fang et al. 2019; Wang et al. 2017). Personalised fracture plates offer key advantages compared to standard off-the-shelf plates. Personalised fracture plates may be designed to precisely follow an individual’s bony anatomy and allow for increased control of screw placement and trajectories by enabling custom positioning of screw holes. Additionally, personalised fracture plates avoid the need for intraoperative contouring of fracture plates, which is imprecise, time-consuming, and may weaken the fatigue properties of the metal (Wang et al. 2017). Pelvic ring fractures are well suited to the use of personalised fracture plates, as the complex anatomy of the pelvis often involves irregular fracture patterns that are challenging to access surgically (Wang et al. 2020, 2017). Moreover, their relatively low incidence (Gabbe et al. 2011) has led to a paucity of off-the-shelf components to treat this anatomical region.

To design a patient-specific plate for a pelvic ring fracture, a 3D model of the unfractured hemipelvis is needed to produce a plate that secures the bone fragments in their original positions, which closes the fracture and facilitates bone healing (Sarkalkan et al. 2014; Wang et al. 2017). This process of reducing the fracture begins by segmenting major bone fragments from 3D medical imaging, typically computed tomography (CT), which may be challenging when fracture boundaries are poorly defined (Paulano et al. 2014). The pieces are subsequently aligned in their original position, either by manual translation and rotations, or using automated alignment tools (Boudissa et al. 2015; Ead et al. 2020b; Zeng et al. 2016). While these approaches have achieved satisfactory results (Zeng et al. 2016), the time-consuming nature of the segmentation and alignment process presents a key bottleneck in the personalised fracture design workflow. Given the time-critical constraints involved in the clinical treatment of pelvic fractures (Giannoudis and Pape 2004; Halawi 2016; Katsoulis and Giannoudis 2006), methods that automatically reconstruct the bone geometry are needed to expediate the design of patient-specific fracture plates within a reasonable timeframe (i.e. within one week; Halawi 2016; Katsoulis and Giannoudis 2006).

A method that has been used to rapidly calculate the unfractured bone geometry following a fracture is using statistical shape models (SSMs) (Ead et al. 2020b), which represent geometric variations of an object across a population as a linear combination of principal mode shapes. By establishing an SSM from a training set of unfractured bones, it may be fitted onto a fragment of bone via a superposition of its principal mode shapes. SSMs have been shown to reconstruct bones such as the femur based on partial surface data with a root-mean-squared error (RMSE) less than 2 mm if more than 90% of the surface is available (Nolte and Bull 2019; Zhang and Besier 2017), and guide the positions of bone fragments with an RMSE of 1.46 ± 0.32 mm (Ead et al. 2020b).

For bilateral bones, mirroring of the contralateral anatomy is a common approach used to guide bone fragment alignment when reconstructing bone fractures (Ead et al. 2020c). Rather than reassembling the bone fragments, the geometry of the mirrored contralateral bone had been used to represent the ipsilateral bone in its unfractured state (Fang et al. 2019). In either case, for mirroring techniques to be effective, symmetry must exist between each side of the body, which has been found to depend on sex and biomechanical factors (Auerbach and Ruff 2006). For the pelvis, previous studies have shown that pairs of hemipelves have a root-mean-square difference of 1.14 ± 0.26 mm (Ead et al. 2020a), which is likely the limit in accuracy that may be achieved using the contralateral mirroring approach. Compared to SSMs, contralateral mirroring has been shown to more accurately reconstruct the femur in cases where the ipsilateral femur comprised of less than 90% of its surface geometry (Nolte and Bull 2019). Contralateral mirroring and SSMs were also found to similarly reconstruct the hemipelvis following tumour resection, with a mean difference of 0.255 mm (Krol et al. 2013). Despite the apparent similarity, no previous studies have compared the accuracy between contralateral mirroring and SSMs for reconstructions of the pelvis, particularly for different pelvic regions and landmarks.

SSMs and contralateral mirroring each offer a rapid method to determine unfractured bone geometries without the need to segment each bone fragment and manually reassemble them in position. However, it is unknown how accurately each of these methods compare for reconstructing pelvic regions and landmarks based on incomplete amounts of surface data. The aim of this study, therefore, was to compare the ability of SSMs and contralateral mirroring to reconstruct the hemipelvis based on partial surface data. The authors hypothesised that contralateral mirroring would offer a reconstruction of similar accuracy to SSMs when data are sparse. By quantifying the ability of these methods to reconstruct the pelvis with varying amounts of surface data, it will be possible to establish under which scenarios each method is more effective. This information is necessary for the improvements of algorithms that reconstruct pelvic bones for the design of personalised fracture plates.

## Methods

### Subject information

Left and right hemipelves were segmented from CT scans of 33 females [age 49.7 ± 14.4 years (mean ± 1SD)] obtained from the Cancer Imaging Archive (Holger et al. 2015; Roth et al. 2014) with an average voxel size in the axial plane of 0.63–0.98 mm and a slice increment of 1 mm. The right hemipelves of two specimens were omitted from the dataset due to poor imaging that prevented proper segmentation of the full pelvis, leaving 64 hemipelves total. Following 3D reconstruction, noise removal, and basic smoothing, a triangular surface mesh was generated with an average of 94,464 polygons with an average length of 2 mm.

### Sparse surface geometry

**Fig. 1:**
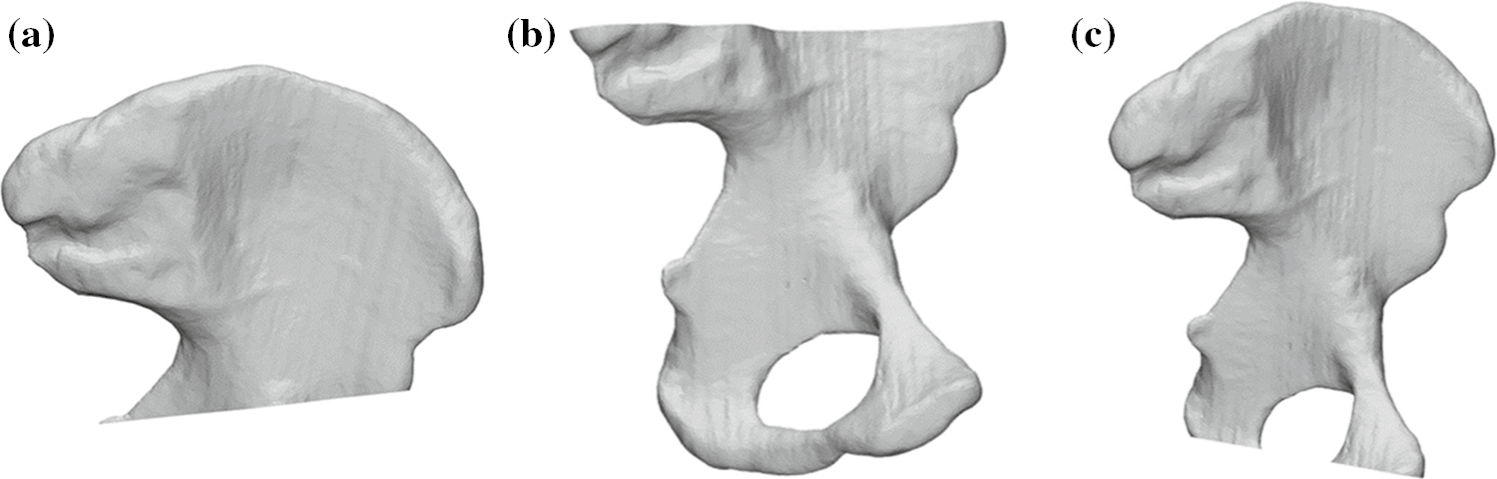
Virtual cuts made to the hemipelvis to simulate sparse data. IS cut **a**: from the tip of the ischial spine protrusion (IS) to two points on the lateral and medial margin of the anterior inferior iliac spine (AIIS). Ilium cut **b**: from the posterior superior iliac spine (PSIS) to two points on the lateral and medial margin of the anterior superior iliac spine (ASIS). PT cut **c**: from the pubic tubercle (PT) to the medial and lateral position of the thickest area of the ischium

To represent sparse geometric data, the surface geometry of each left hemipelvis was cut by a plane that passed through a set of pelvic landmarks. Three cuts were considered (Fig. 1): (1) IS cut: from the tip of the ischial spine protrusion (IS) to two points on the lateral and medial margin of the anterior inferior iliac spine (AIIS), (2) ilium cut: from the posterior superior iliac spine (PSIS) to two points on the lateral and medial margin of the anterior superior iliac spine (ASIS), and (3) PT cut: from the pubic tubercle (PT) to the medial and lateral position of the thickest area of the ischium. As there are many different forms of pelvis fractures, the cuts were selected such that they could be repeatably performed using pelvic landmarks while offering fractures of differing severities at three different locations. For example, the IS cut mimics a hemipelvis which has had data omitted around the acetabulum and inferior pelvis as might be expected in the case of an acetabular or pubic ramus fracture. Once cut, the open surface was closed by a flat fill across the cutting plane. The IS, ilium, and PT cuts reduced the surface to 68%, 75%, and 94% of the original hemipelvis surface area, respectively.

### Contralateral mirroring and statistical shape models

For contralateral mirroring, each of the 31 right hemipelves was mirrored about the sagittal plane. The software GIAS2, which is part of the open-source platform, the MAP Client (J. Zhang et al. 2014a), was used to establish mesh correspondence and align all hemipelves. To establish mesh correspondence, a representative left hemipelvis of average height and width was selected as a hostmesh and each hemipelvis was non-rigidly registered to the hostmesh using radial basis functions. Once correspondence was established, the fitted meshes were rigidly aligned to the hostmesh using Procrustes analysis (Stegmann and Gomez 2002) to remove any rotational and translational variation.

The SSMs were then generated by a principal component analysis to quantify the principal modes of variation across the training set that comprised the 33 left hemipelves and 31 mirrored right hemipelves.

The SSM was evaluated using a leave-one-out cross validation strategy (J. Zhang et al. 2014b), where the SSM was fitted for each case to a paired left hemipelvis omitted from the training set. The omitted hemipelvis was reconstructed by morphing the generated SSM using a selected number of principal components. The number of principal components was selected by first reconstructing hemipelves from intact input data using a range of principal components and then selecting the number of principal components which minimised root-mean-squared error (RMSE). Optimal fitting was achieved by minimising the least-squares distance between the target and the fitted SSM surface points (Zhang and Besier 2017). This process was repeated for the four SSM reconstructions (i.e. the intact geometry and the sparse surface geometry with the three different cuts) for each of the 31 paired left hemipelves.

### Landmarks and region assignment

**Fig. 2:**
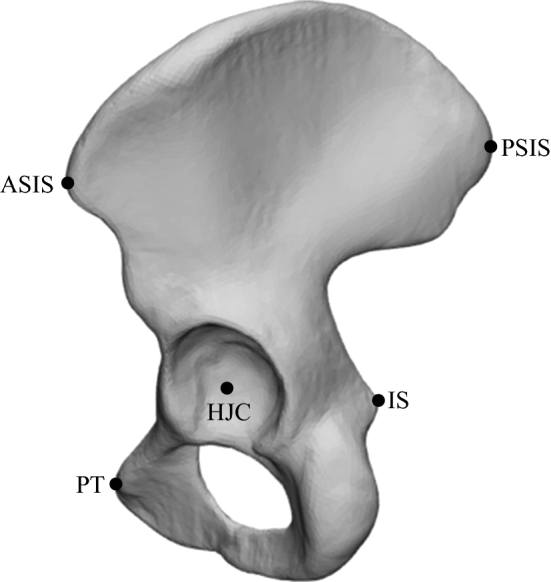
Location of landmarks used (anterior superior iliac spine (ASIS), posterior superior iliac spine (PSIS), ischial spine protrusion (IS), pubic tubercle (PT), hip joint centre (HJC) on a representative hemipelvis

Surface regions containing nodes and polygons were manually identified in the hostmesh for the acetabulum, ilium, ischium, and pubis regions of the hemipelvis. When establishing mesh correspondence or during SSM fitting, these nodes and polygons were assumed to remain within their respective anatomical regions. This assumption was not applicable for pelvic landmarks, as polygons easily shifted outside of the small anatomical region of the landmark during the non-rigid registration. Hence, pelvic landmarks were manually identified for all hemipelves by selecting a small surface region at the respective landmark position. Pelvic landmarks included the ASIS, PSIS, IS, and PT (Fig. 2). To account for any variations in triangle sizes, the centroid of each landmark was calculated using an area-weighted method:

1 $${\text{centroid}} = { }\frac{{\sum {\text{area}}\left( t \right)}}{{\sum \left( {{\text{area}}\left( t \right){\text{*centroid}}\left( t \right)} \right)}}$$

where $t$ is each triangular element comprising the landmark region.

An additional landmark, the hip joint centre (HJC), was defined as the centre of a sphere fitted to the acetabulum region of the hemipelvis.

### Statistical analysis

The accuracy of the SSM and contralateral mirroring methods to reconstruct the original hemipelvis was quantified by first using correspondent nodes to identify and align pelvic regions and landmarks. An RMSE was then calculated as the Euclidean distance between the aligned regions and landmarks. Significant differences in the RMSE and landmark deviation between the SSM data and contralateral mirroring were determined using paired t tests, with statistical significance considered for *p* < 0.05.

## Results

After fitting the SSM to the intact hemipelvis for a varying number of principal components, it was found that 30 principal components produced the lowest average RMSE. This number of principal components was used for all SSM reconstructions in the current study. When fit to an intact hemipelvis, the RMSE of the SSM (1.11 ± 0.54 mm) had a lower mean value than for contralateral mirroring (1.21 ± 0.29 mm); however, this difference was not significant (*p* > 0.05) (Table 1 and 2). There were also no significant differences between the methods for the acetabulum and ilium regions. For the ischium, however, the RMSE for contralateral mirroring (0.83 ± 0.20 mm) was significantly lower than the SSM (0.97 ± 0.19 mm) (*p* < 0.05). Similarly, for the pubis the RMSE for contralateral mirroring was significantly lower than the SSM (0.85 ± 0.24 mm and 0.97 ± 0.23 mm, respectively) (*p* < 0.05).

**Table 1:** Mean RMSE (mm) and standard deviation of RMSE (mm) of hemipelves reconstructed via contralateral mirroring and shape modelling

Reconstruction model and geometry		Percentage of original surface (%)	Whole hemipelvis		Acetabulum		Ilium		Ischium		Pubis	
			Mean	SD	Mean	SD	Mean	SD	Mean	SD	Mean	SD
Contralateral mirroring		N/A	1.21	0.29	0.73	0.20	1.11	0.23	0.83	0.20	0.85	0.24
SSM	Intact hemipelvis	100	1.11	0.54	0.75	0.12	1.07	0.49	0.97	0.19	0.97	0.23
	IS cut	68	1.83	0.85	0.83	0.17	1.43	0.77	1.23	0.24	1.22	0.28
	Ilium cut	75	1.51	0.62	0.76	0.11	1.47	0.50	1.02	0.22	1.02	0.25
	PT cut	94	1.25	0.76	0.77	0.15	1.13	0.59	1.05	0.22	1.06	0.29

*IS*, ischial spine protrusion; *PT*, pubic tubercle

**Table 2:** *P*-values from two-sided t-test comparing mean RMSE values from contralateral mirroring to that of each shape model reconstruction across each region

Reconstruction geometry	Pelvic region				
	Whole hemipelvis	Acetabulum	Ilium	Ischium	Pubis
Intact hemipelvis	0.32	0.46	0.65	< 0.001	< 0.01
IS cut	< 0.001	0.01	0.03	< 0.001	< 0.001
Ilium cut	0.02	0.34	< 0.001	< 0.001	< 0.01
PT cut	0.82	0.29	0.92	< 0.001	< 0.001

*IS*, ischial spine protrusion; *PT*, pubic tubercle

**Fig. 3:**
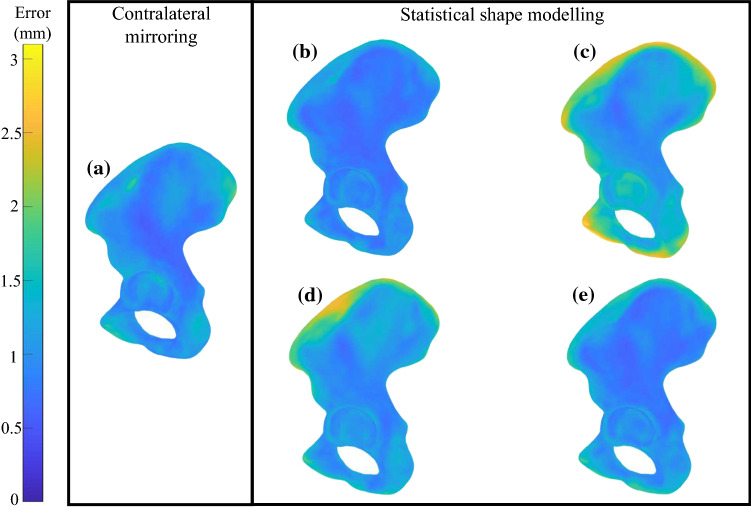
Contour plots showing average error (mm) at every element for the contralateral hemipelvis (**a**), and the SSM reconstructions based on the whole hemipelvis (**b**), the IS cut (**c**), the ilium cut (**d**), and the PT cut (**e**)

Reconstructions of the hemipelvis based on partial surface data indicated that the RMSE computed across the whole hemipelvis for contralateral mirroring (1.21 ± 0.29 mm) was significantly lower than the SSM for the IS cut (1.83 ± 0.85 mm) and the ilium cut (1.51 ± 0.62 mm) (each *p* < 0.05; Table 1, Fig. 3). When considering the ischium and pubis regions, the RMSE for contralateral mirroring was significantly lower than the SSM for all three cuts (each *p* < 0.05). For the ilium, the RMSE for contralateral mirroring was significantly lower than the SSM for the IS cut and ilium cut (each *p* < 0.05). For the acetabulum, the RMSE for contralateral mirroring was only significantly lower than the SSM for the IS cut (*p* < 0.05). For all other regions and cuts, there was no significant difference between the contralateral mirroring and the SSM-based reconstructions.

Considering the landmarks (Tables 3 and 4), the only significant difference was noted for the PT landmark, where the deviation for contralateral mirroring (0.83 ± 0.93 mm) was significantly lower than the SSM when fit to the intact hemipelvis (1.02 ± 0.84 mm), the ilium cut (1.03 ± 0.78 mm), and the PT cut (1.11 ± 0.84 mm) (*p* < 0.05 each).

**Table 3:** Mean landmark deviations (mm) and standard deviation of landmarks (mm) of reconstructed hemipelves

Reconstruction model and geometry		Percentage of original surface (%)	ASIS		PSIS		IS		PT		HJC	
			Mean	SD	Mean	SD	Mean	SD	Mean	SD	Mean	SD
Contralateral mirroring		N/A	1.51	0.57	0.83	1.46	0.31	0.77	0.83	0.93	0.90	0.61
SSM	Intact hemipelvis	100	0.88	0.56	1.65	1.48	1.13	0.75	1.02	0.84	0.86	0.68
	IS cut	68	0.93	0.47	1.61	1.47	1.16	0.80	1.22	0.97	1.02	0.84
	Ilium cut	75	0.90	0.52	1.62	1.54	1.26	0.97	1.03	0.78	0.89	0.67
	PT cut	94	0.88	0.57	1.67	1.53	1.12	0.73	1.11	0.84	0.90	0.70

*ASIS*, anterior superior iliac spine; *PSIS*, posterior superior iliac spine; *IS*, ischial spine protrusion; *PT*, pubic tubercle; *HJC*, hip joint centre

**Table 4:** *P*-values from two-sided t-test comparing landmark deviation of contralateral mirroring to that of each SSM reconstruction

Reconstruction geometry	Pelvic landmark				
	ASIS	PSIS	IS	PT	HJC
Intact hemipelvis	0.83	0.99	0.93	< 0.001	0.60
IS cut	0.64	0.72	0.83	0.42	0.46
Ilium cut	0.95	0.84	0.31	< 0.01	0.84
PT cut	0.89	0.78	0.87	0.02	0.90

*ASIS*, anterior superior iliac spine; *PSIS*, posterior superior iliac spine; *IS*, ischial spine protrusion; *PT*, pubic tubercle; *HJC*, hip joint centre

## Discussion

This work quantified the ability of contralateral mirroring and SSMs to reconstruct the surface of the hemipelvis based on partial surface geometries. Regardless of the amount of surface geometry available for fitting the SSM, contralateral mirroring represented the hemipelvis geometry with an RMSE that was either significantly lower or statistically equivalent when using SSMs (Tables 1 and 2). The ischium and pubis regions generated via contralateral mirroring had significantly lower RMSE (*p* < 0.05) than the SSM reconstructions that were fitted to the intact hemipelvis (Tables 1, 2). This result indicates a high degree of variability in these regions that may be represented by the symmetry between the left and right sides of the pelvis, but poorly captured using an SSM, at least with a training set based on 64 hemipelves.

When reconstructing the hemipelvis geometry based on the partial surface geometry corresponding to the IS cut, all pelvic regions were reconstructed with significantly lower RMSE using contralateral mirroring compared to the SSM (Table 2). For the ilium and PT cuts, some of the pelvic regions had significantly lower RMSE for contralateral mirroring compared to the SSMs, such as the ischium and pubis regions, and there was no situation where the SSM provided a significantly lower RMSE compared to the contralateral mirroring. The deviations of the landmarks were not significantly different between each reconstruction method, apart from the PT landmark which was significantly lower for the contralateral mirroring compared to SSMs when reconstructed from the intact hemipelvis, and ilium and PT cut scenarios (Table 4). These results strongly support the use of contralateral mirroring compared to the use of SSMs in all scenarios of pelvic bone reconstructions.

The RMSEs obtained in the current study compare well to previous work examining contralateral mirroring or SSMs for pelvis reconstruction. For instance, the RMSE obtained for the full hemipelvis with contralateral mirroring was 1.21 ± 0.29 mm (Table 1), which is comparable to the RMSE of 1.14 ± 0.26 mm reported by (Ead et al. 2020b). The RMSE calculated over the entire hemipelvis based on the ilium cut SSM reconstruction was 1.51 ± 0.62 mm (Table 1), which is comparable to the RMSE reported by Ead et al. 2020a, b, c, for similar fractures with 1.46 ± 0.32 mm (Ead et al. 2020b).

The similarities between SSMs and contralateral mirroring observed in the current study are in agreement with previous work focused on hemipelvis reconstructions, where each method generated surfaces that differed by a mean of 0.255 mm (Krol et al. 2013). While Krol et al. reported an RMSE for the SSMs (1.26 ± 1.08 mm) that was comparable to the current study (1.11 ± 0.54 mm), these authors did not provide the RMSE for contralateral mirroring which limits a detailed comparison between the two reconstruction techniques. Moreover, these authors did not examine the effects of varying surface information as examined in the current study.

**Fig. 4:**
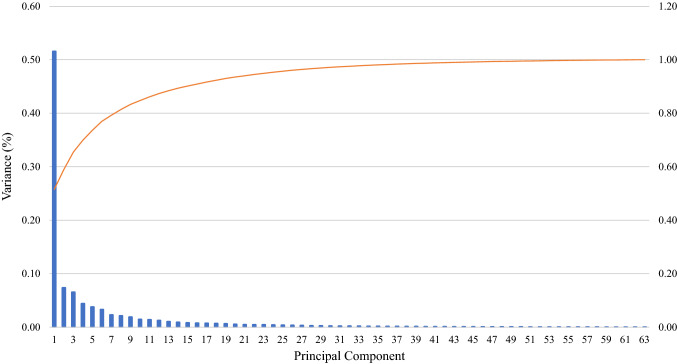
Plot of percentage variation explained by individual principal components (blue) and cumulative variation (orange) explained by principal components for SSM generated from all hemipelves

Another study that analysed reconstructions of the femur reported that the RMSE for contralateral mirroring was significantly lower or equivalent to SSMs when less than 90% of the surface geometry was available (Nolte and Bull 2019). This was similar to the current study where the RMSE for reconstructing the whole hemipelvis using contralateral mirroring was significantly lower than SSMs for the IS cut (*p* < 0.001) and ilium cut (*p* = 0.02) that had 68% and 75% of the surface information available, respectively. However, for reconstructions based on greater than 90% of the bone geometry, Nolte & Bull found that the RMSE from the SSM reconstructions was significantly lower than the contralateral mirroring (*p* < 0.001), a finding that was not observed in the current study. This difference may be related to a greater extent of anatomical variation in the pelvis compared to the femur. This is demonstrated by the SSM compactness, a measure of the number of principal components needed to describe a fixed percentage of variation. Thirty principal components were required to describe 97% of the variance of the hemipelvis surface (Fig. 4), while only five principal components were needed to describe 99% of femoral surface variance (Zhang et al. 2016).

The results of the current study may be limited by the training set which was sourced from the Cancer Imaging Archive, involving patients with lymphadenopathy (i.e. enlarged lymph nodes) of the mediastinum or abdomen (Holger et al. 2015; Roth et al. 2014). Despite no apparent abnormalities present in the pelvic bone reconstructions, it cannot be conclusively verified whether the bones represented healthy morphology unaffected by cancer. The size of the training set in the current study (*n* = 64) is also a valid concern as it was lower than previous studies, such as 110 femora in Zhang and Besier (2017) and 542 hemipelves used by Audenaert et al. (2019); however, it was still larger than the 40 hemipelves used by Ead et al. (2020b), 40 femora used by Nolte and Bull (2019), and 50 hemipelves used by Krol et al. (2013). Additionally, the 64 hemipelves were gathered from 33 subjects. While the inclusion of both left and right hemipelves of these subjects likely captures less of the variability present in the wider population, the symmetry included in the training set should assist the SSMs to reconstruct the original geometry since the mirrored contralateral hemipelvis included in the training set had some geometric similarity to the ipsilateral hemipelvis. While a larger training set would encompass a greater range of anatomical variability that may increase the accuracy of the SSM, the RMSE of the SSM fitted to the full hemipelvis was 1.11 ± 0.54 mm, which is similar to the previous studies for the femur (1.57 ± 0.49 mm; J. Zhang et al. 2014b) and hemipelvis (1.26 ± 1.08 mm; Krol et al. 2013). Instead, the accuracy is most likely limited by the CT scan resolution, which was 1 mm in the current study. Hence, it was considered unlikely that a larger training set would significantly alter the results in the current study.

Sparse hemipelvis geometry was generated by making uniform cuts made between pelvic regions and landmarks. While these simplified cuts were not representative of fracture pathologies, they were readily applied to pelvic landmarks, which provided a repeatable method for all pelvic geometries. Additionally, these cuts encompassed all three bones within the hemipelvis (i.e. ilium, ischium, and pubis), thereby allowing the reconstructive performance of the SSM to be determined for potential fracture locations across the hemipelvis.

## Summary

In summary, contralateral mirroring provides a rapid technique for hemipelvis reconstruction that produces an RMSE significantly lower or statistically equivalent to SSMs across all regions and landmarks evaluated. This is in partial agreement with the hypothesis that contralateral mirroring would offer a similar reconstruction accuracy when reconstructing hemipelves from sparse data, as contralateral mirroring was found to be at least as accurate as SSMs for this application. However, in cases where asymmetry between hemipelves is apparent, or pathologies such as bilateral fractures, deformities, or metastasis make the contralateral hemipelvis unavailable for mirroring, SSMs provide a viable reconstruction method of reasonable accuracy depending upon the amount of hemipelvis surface information available. This study quantifies the relationship between SSM reconstruction accuracy and the available proportion of the hemipelvis surface, information that may be used to evaluate when manual reconstruction procedures are warranted due to insufficient surface information. The results of this work inform the development of algorithms that automatically reconstruct pelvic fractures, which will facilitate the rapid design of personalised fracture plates that conform to patient-specific anatomy.

## Data Availability

Data may be made available on request.

## Code availability

Custom code was used to perform RMSE evaluation of hemipelvic models.
